# Representativeness of participants in the Danish National Health Survey across 422,371 orthopedic surgeries: a study of hip and knee arthroplasty and hip fracture patients

**DOI:** 10.1007/s00402-025-05924-7

**Published:** 2025-05-27

**Authors:** Simon Storgaard Jensen, Nadia R. Gadgaard, Heidi Amalie Rosendahl Jensen, Lei Wang, Alma Becic Pedersen

**Affiliations:** 1 Department of Clinical Epidemiology, Aarhus University Hospital, Olof Palmes Allé 43-45, Aarhus N, 8200 Denmark; 2 Department of Clinical Medicine, Aarhus University, Aarhus, Denmark; 3 National Institute of Public Health, University of Southern Denmark, Copenhagen, Denmark

**Keywords:** Surgery, Arthroplasty, Prosthesis, Hip, Knee, Fracture

## Abstract

**Aims:**

Orthopedic registries have provided valuable knowledge about risk for and prognosis after total hip arthroplasties (THA), knee arthroplasties (KA), and hip fractures. However, registries are often limited by the lack of data on health risk behaviors, quality of life, and social background, which are readily available in surveys. We examined if participants in The Danish National Health Survey, based on self-administered questionnaires, are representative of THA, KA, and hip fracture patients.

**Methods:**

Patients were identified in the Danish orthopedic registries and linked with survey data (from 2010, 2013, and 2017) on an individual level. Data on age, sex, comorbidity, medication, markers of socioeconomic position, and healthcare utilization were assessed from the Danish medical databases. We calculated the proportions of variables before and after surgery, comparing patients who had and had not participated in surveys.

**Results:**

We included 177,617 THA surgeries (4.5% of patients completed pre-surgery surveys and 7.0% completed post-surgery surveys), 152,154 KA surgeries (7.0% of patients completed pre-surgery surveys and 6.2% completed post-surgery surveys) and 92,600 hip fracture surgeries (3.8% of patients completed pre-surgery surveys and 2.2% completed post-surgery surveys). Survey participants and non-participants had similar age and sex distribution in the three cohorts. Based on comorbidity, medication, and healthcare utilization, participants appeared slightly healthier than non-participants. There was a slight variation in socioeconomic markers for THA and KA patients between participants and non-participants.

**Conclusion:**

The Danish National Health Survey provides a sample that appears to be largely representative of all THA, KA, and hip fracture patients in Denmark. Survey data could be a valuable data source for further studies of the risks and outcomes associated with patients undergoing THA and KA and those suffering from hip fractures, while carefully considering the identified similarities and differences when designing studies and analyzing the survey data.

**Supplementary Information:**

The online version contains supplementary material available at 10.1007/s00402-025-05924-7.

## Introduction

The establishment of national orthopedic registries started in Scandinavian countries in the early seventies, aiming to monitor and improve the quality of patient treatment continuously [1, 2]. These registries have proven valuable by providing data for large and high-quality studies about the risk for and prognosis following orthopedic procedures [3–5]. Orthopedic registries contain data on patient characteristics, surgical information, implants, process performance measures, and outcome data, including reoperation and mortality [3, 4, 6]. The completeness and data validity of Danish arthroplasty and hip fracture registries are considered high [3, 4, 6, 7].

In recent years, patient-reported outcome measures (PROMs) were added to several orthopedic registries [8–11], acknowledging the need for patient perspectives to improve patient-centered care [12, 13]. However, orthopedic registries are still limited by the lack of more detailed data on health risk behaviors, health perception, morbidity, and social support, all of which could be related to patient outcomes. In particular, self-reported pain, need for assistance, and daily activity limitations might be highly relevant for defining a successful outcome.

The Danish National Health Surveys are nationwide cross-sectional health surveys of the general population in Denmark aged 16 years or older. The surveys, which are based on data from self-reported questionnaires, have been conducted in 2010, 2013, 2017, and 2021 [14]. The objective of the surveys is to describe the status and trends in health, health behaviors, and morbidity in the population to provide data to promote better health through targeted prevention and interventions. The survey data are available for research [14, 15]. The survey questions revolve around self-rated lifestyle factors, health-related quality of life, health behavior, morbidity, and social relations. The surveys have contributed to answering multiple research questions in various settings [16–18].

Given the scarcity of self-reported measures in orthopedic research, the Danish National Health Surveys could serve as a valuable data source. However, it is largely unknown whether survey participants are representative of orthopedic patients, as the survey samples include only a random sample of individuals with a permanent residence in Denmark. We examined whether participants in the Danish National Health Surveys were representative of all patients in Denmark undergoing total hip and knee arthroplasty and hip fracture patients as registered in the Danish Hip and Knee Registry and the Danish Multidisciplinary Hip Fracture Registry.

## Materials and methods

Healthcare in Denmark is administratively and geographically divided into five administrative regions, which provide universal tax-financed healthcare services to all residents with access to primary and secondary care [19]. Each citizen has a unique social security number assigned at birth or immigration, enabling accurate linkage between all registries at an individual level [19, 20]. Furthermore, the social security number enables unique linkage between nationwide surveys like the Danish National Health Surveys and registries.

### Study population

The survey population was derived from the Danish National Health Surveys conducted in collaboration with the Danish Health Authority, the National Institute of Public Health, and the five regions of Denmark every fourth year [15]. The surveys are based on six mutually exclusive random subsamples, one from each of the five regions and one additional national sample of people aged 16 or older who were invited to participate in the study. The self-administered questionnaires were sent electronically by a secure e-mail service or by post to invite individuals who had actively deregistered from this service. Up to three reminders were sent if people did not complete and return the questionnaire. The questionnaire contains questions on several health-related topics, e.g., stress, pain, diseases and complications, sleep, smoking, alcohol consumption, dietary habits, physical, family and social situation, and employment [14].

The questionnaire was fully or partially completed by 117,639 (59.5%), 162,283 (54.0%), and 183,372 (58.7%) invited individuals in 2010, 2013, and 2017, respectively. Due to the age distribution of hip and knee arthroplasty patients, we restricted survey data to patients aged 35 years or older. Due to the definition within the hip fracture register, patients within this register are aged 65 or older. Thus, this study included a total number of 411,987 survey respondents.

Upon survey participation, the respondents were linked on an individual level to the Danish Hip Arthroplasty Register (DHR) [4], the Danish Knee Arthroplasty Register (DKR) [6], and the Danish Multidisciplinary Fracture Registry (DMHFR) [3] to identify individuals who underwent primary total hip arthroplasty (THA), knee arthroplasty (KA), or hip fracture surgery. The data included patients who had surgery from DHR (1995–2018), DKR (1997–2021), and DMHFR (2004–2018). In the case of bilateral THA or KA, both surgeries were included, while only first-time unilateral hip fracture surgery was included, corresponding to the definitions of the target populations in the respective registries.

**Fig. 1:**
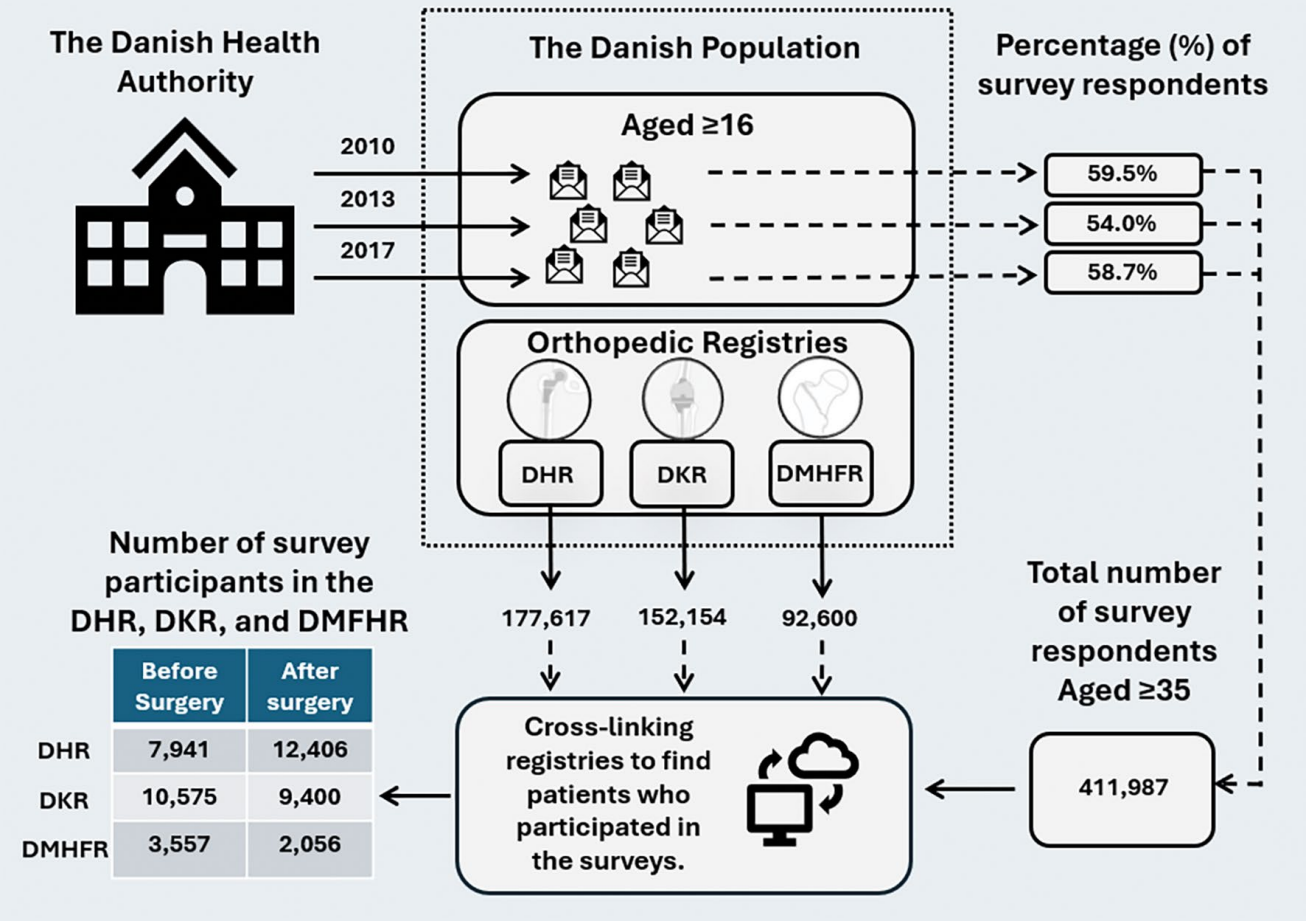
Linkage between the National Danish Health Surveys and Danish Hip Arthroplasty Register (DHR), the Danish Knee Arthroplasty Register (DKR), and the Danish Multidisciplinary Fracture Registry (DMHFR)

The linkage between survey data and DHR, DKR, and DMHFR was done on the surgery date, allowing us to capture survey participation both before and after surgery. If a patient participated in the survey on multiple occasions before and after surgery, every participation was counted. By cross-referencing survey data with the DHR, DKR, and DMHFR, we identified patients who participated in the survey and the corresponding year of participation. For those who did not participate, it was unclear whether they were not invited or simply chose not to respond. Therefore, patients were categorized as either participants or non-participants, rather than respondents or non-respondents (Fig. 1).

We defined three separate subpopulations of primary THA, KA, and hip fracture patients. Afterward, patients were analyzed and stratified by survey participation status, i.e., whether they were survey participants or non-participants at the time of their surgery date.

## Variables

To compare subpopulations and examine their representativeness, we included the following variables from national medical databases for each patient at the date of THA, KA, or hip fracture surgery:

1. Patient characteristics (age, sex, cohabitation status, and area of residence) were collected from the Civil Registration System [19, 20].
2. Comorbidities were obtained from the Danish National Patient Register [21]. Based on the International Classification of Diseases 10th revision (ICD-10) codes, information on all 19 diagnoses included in the Charlson Comorbidity Index [22] (CCI) was collected with a 10-year lookback period. Both primary and secondary diagnoses, as well as diagnoses from in-hospital and outpatient visits, were used. We calculated the CCI score and defined three levels of comorbidity: a CCI score of 0 (low) for patients with no previous record of the diseases included in the index; a CCI score of 1–2 (medium); and a CCI score of 3 or more (high). In addition, we included information on mental disorders and several additional relevant somatic comorbidities, extending the CCI with the Elixhauser Index and Rx-Risk Index, based on previous literature and their prevalence within the cohorts [23] (Supplementary Appendix A).
3. Medication use one year before the surgery date was obtained from the Danish National Prescription Register [24]. We included information on common prescriptions for comorbidities and the Rx-Risk Index: Oral anticoagulants, antidepressants, antidiabetics, antihypertensive drugs, anti-thyroid medication, antipsychotics, antithrombotics, anxiolytics and sedatives, chronic obstructive pulmonary disease medication, corticosteroids, hormone replacement therapy, non-steroid anti-inflammatory drugs, opioids, osteoporosis-related medication, and statins (Supplementary Appendix B).
4. Socioeconomic factors: We obtained individual-level information on education and income from the Population Education Register and the Income Statistics Register in Denmark Statistics [19, 20]. We categorized education based on the highest obtained education: low (high school or less), medium (vocational education or higher general and preparatory examination programs), high (bachelor’s degree or higher), or missing (no registered education). To account for yearly income variation, we calculated the mean annual household income over the five calendar years before surgery and divided it into four equally large groups.
5. Health care utilization in the year before surgery was obtained from the Danish National Patient Registry [21]. We counted the number of hospitalizations, outpatient visits, and emergency room visits.

Surgery data was obtained from DHR, DKR, and DMHFR [3, 4, 6] including the date of surgery, time from the survey completion date to the surgery date, and time from the surgery date to the survey completion date.

### Statistical analysis

We calculated the prevalence and prevalence ratios comparing patients who had participated and those who had not participated in surveys before or after the surgery date for various variables for all subpopulations. For example, we calculated the prevalence of females among primary THA patients who were survey participants and non-participants before and after the surgery date.

All data were analyzed and managed using SAS software (SAS 9.4).

### Patient involvement

Patients were neither involved in formulating the research question or outcome measures nor did they participate in the design or implementation planning of the study. Additionally, no patients were consulted for interpretation or writing of the results. Patients received an introductory letter, where it was emphasized that participation was voluntary, consent for participation, and individual anonymization [14].

## Results

### Description of THA surveys participants and non-participants

Among the 177,617 THA surgeries identified in the DHR, 7,941 (4.5%) patients participated in surveys before THA, and 12,406 (7.0%) participated in surveys after THA. The median time was 3.5 years from survey to THA among participants before THA and 5.1 years from surgery to survey among participants after THA.

**Fig. 2:**
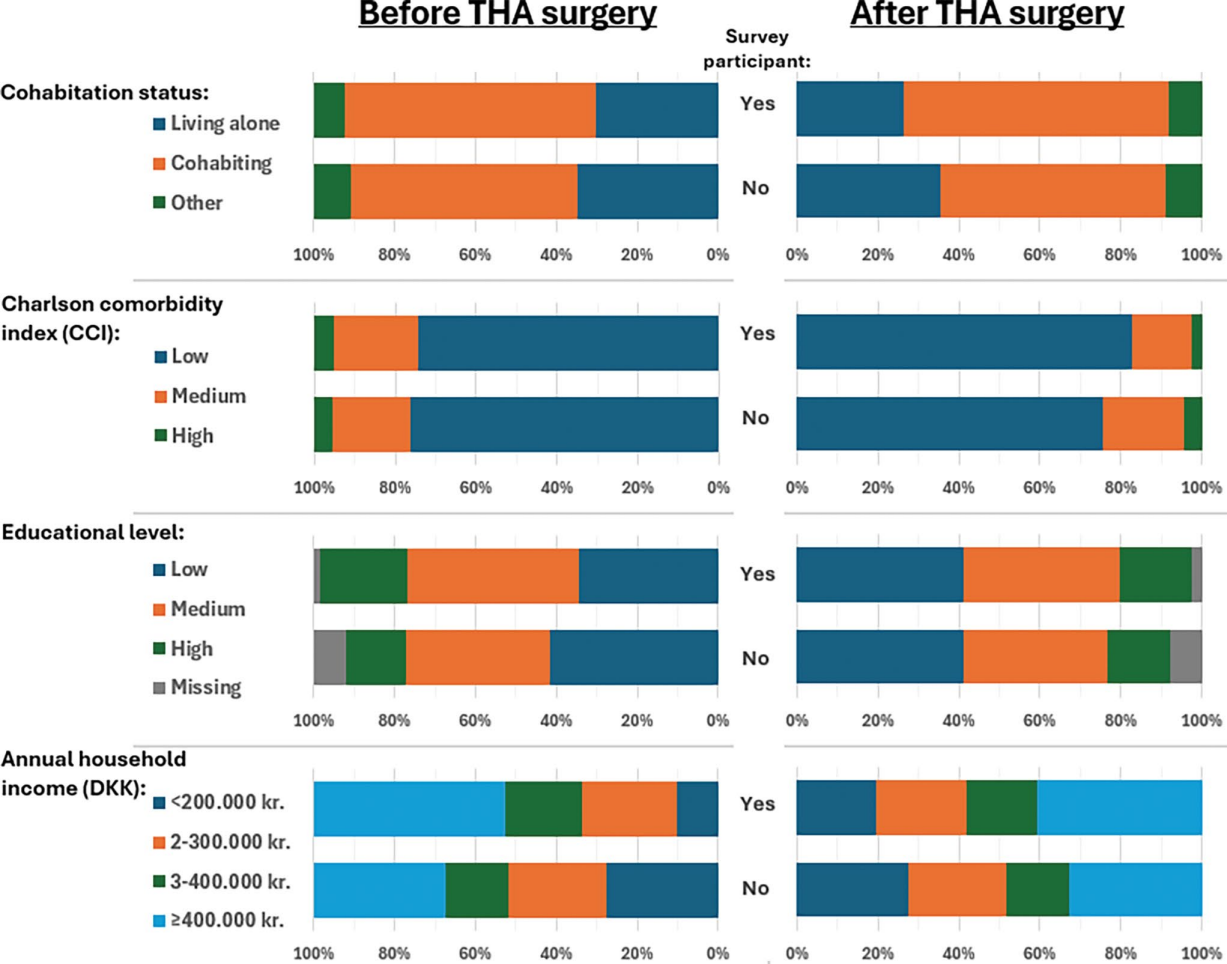
Prevalence and distribution of socioeconomic position and markers Charlson Comorbidity Index among hip arthroplasty patients (THA)* based on survey participation and the index surgery date. Exact numbers (n) and percentages (%) are provided in Supplementary Table 1. *As recorded in the Danish Hip Arthroplasty Registry. Abbreviation: DKK, The Danish krone

Before THA, similar sex distribution (58% females) and median age (70–71 years) were observed among THA survey participants and non-participants. After THA, the prevalence of females among participants was 54% with a median age of 67 years, whereas the prevalence of females among non-participants was 58% with a median age of 70 years. (Table 1). Before THA, 30% of survey participants were living alone, compared to 35% among non-participants. After THA, 26% of survey participants were living alone, compared to 35% of non-participants (Fig. 2).

**Table 1:** Prevalence and prevalence ratios (PR) for various variables among hip arthroplasty patients (THA)* based on survey participation and the index surgery date

	Before THA surgery			After THA surgery		
	Survey participants (*n* = 7,941)	Non-survey participants (*n* = 169,676)	PR (95% CI)	Survey participants (*n* = 12,406)	Non-survey participants (*n* = 165,211)	PR (95% CI)
**Survey/surgery, years median**						
Survey to surgery	3.45	n/a	n/a	2.34	3.61	n/a
Surgery to survey	2.15	5.31	n/a	5.12	n/a	n/a
**Patient characteristics**						
Sex, n (%)						
Female	4,525 (56.98)	98,024 (57.77)	0.97 (0.9, 1.0)	6,744 (54.36)	95,805 (57.99)	0.87 (0.8, 0.9)
**Age**,** years median (IQR)**	71.08	70.07	n/a	67.18	70.38	n/a
Age group, n (%)						
35–49	258 (3.25)	8,670 (5.11)	0.63 (0.6, 0.7)	688 (5.55)	8,240 (4.99)	1.11 (1.0, 1.2)
50–54	369 (4.65)	8,833 (5.21)	0.89 (0.8, 1.0)	822 (6.63)	8,380 (5.07)	1.30 (1.2, 1.4)
55–59	562 (7.08)	14,715 (8.67)	0.81 (0.7, 0.9)	1,485 (11.97)	13,792 (8.35)	1.44 (1.4, 1.5)
60–64	944 (11.89)	22,609 (13.32)	0.88 (0.8, 1.0)	2,159 (17.40)	21,394 (12.95)	1.38 (1.3, 1.5)
65–69	1,465 (18.45)	29,542 (17.41)	1.07 (1.0, 1.1)	2,519 (20.30)	28,488 (17.24)	1.20 (1.2, 1.3)
70–74	1,698 (21.38)	31,800 (18.74)	1.17 (1.1, 1.2)	2,245 (18.10)	31,253 (18.92)	0.95 (0.9, 1.0)
75+	2,645 (33.31)	53,507 (31.53)	1.08 (1.0, 1.1)	2,488 (20.05)	53,664 (32.48)	0.54 (0.5, 0.6)
**Region of residence**, **n (%)**						
Capital	2,170 (27.33)	45,311 (26.70)	1.03 (1.0, 1.1)	3,278 (26.42)	44,203 (26.76)	0.98 (0.9, 1.0)
Zealand	1,101 (13.86)	28,649 (16.88)	0.8 (0.8, 0.9)	1,649 (13.29)	28,101 (17.01)	0.76 (0.7, 0.8)
Southern Denmark	1,894 (23.85)	39,976 (23.56)	1.02 (1.0, 1.1)	3,279 (26.43)	38,591 (23.36)	1.16 (1.1, 1.2)
Central Jutland	1,730 (21.79)	38,031 (22.41)	0.97 (0.9, 1.0)	2,525 (20.35)	37,236 (22.54)	0.89 (0.8, 0.9)
Northern Jutland	1,046 (13.17)	17,709 (10.44)	1.28 (1.2, 1.3)	1,675 (13.50)	17,080 (10.34)	1.32 (1.3, 1.4)
**Individual comorbidities**, **n (%)**						
COPD	377 (4.75)	7,599 (4.48)	1.06 (1.0, 1.2)	367 (2.96)	7,609 (4.61)	0.65 (0.6, 0.7)
Chronic renal impairment	110 (1.39)	1,598 (0.94)	1.45 (1.2, 1.8)	59 (0.48)	1,649 (1.00)	0.49 (0.4, 0.7)
Diabetes	803 (10.11)	1,4245 (8.4)	1.22 (1.1, 1.3)	832 (6.71)	14,216 (8.60)	0.78 (0.7, 0.8)
Dementia	63 (0.79)	1,432 (0.84)	0.94 (0.7, 1.2)	26 (0.21)	1,469 (0.89)	0.25 (0.2, 0.4)
Any malignancy	1,225 (15.43)	17,404 (10.26)	1.56 (1.5, 1.7)	918 (7.40)	17,711 (10.72)	0.68 (0.6, 0.7)
Osteoporosis	554 (6.98)	8,151 (4.80)	1.46 (1.3, 1.6)	471 (3.80)	8,234 (4.98)	0.77 (0.7, 0.9)
**Concomitant medication**,** n (%)**						
Oral corticosteroids	750 (9.44)	16,765 (9.88)	0.95 (0.9, 1.0)	1,061 (8.55)	16,454 (9.96)	0.85 (0.8, 0.9)
Antiosteoporosis drugs	768 (9.67)	11,390 (6.71)	1.46 (1.4, 1.6)	665 (5.36)	11,493 (6.96)	0.77 (0.7, 0.8)
Anticoagulants	769 (9.68)	11,323 (6.67)	1.47 (1.4, 1.6)	669 (5.39)	11,423 (6.91)	0.78 (0.7, 0.9)
Antidiabetics	655 (8.25)	11,288 (6.65)	1.25 (1.2, 1.4)	661 (5.33)	11,282 (6.83)	0.78 (0.7, 0.9)
Antithrombotic	1,869 (23.54)	37,632 (22.18)	1.08 (1.0, 1.1)	2,371 (19.11)	37,130 (22.47)	0.83 (0.8, 0.9)
Hormone replacement therapy	1,050 (13.22)	19,610 (11.56)	1.16 (1.1, 1.2)	1,545 (12.45)	19,115 (11.57)	1.08 (1.0, 1.2)
Hormone deprivation therapy	5 (0.06)	197 (0.12)	0.55 (0.2, 1.3)	13 (0.10)	189 (0.11)	0.92 (0.5, 1.6)
Anxiolytics and sedatives	1,131 (14.24)	38,824 (22.88)	0.57 (0.5, 0.6)	2,299 (18.53)	37,656 (22.79)	0.78 (0.7, 0.8)
Antipsychotics	151 (1.90)	5,470 (3.22)	0.59 (0.5, 0.7)	217 (1.75)	5,404 (3.27)	0.54 (0.5, 0.6)
Antidepressants	961 (12.10)	22,140 (13.05)	0.92 (0.9, 1.0)	1,155 (9.31)	21,946 (13.28)	0.69 (0.6, 0.7)
Statins	2,433 (30.64)	34,176 (20.14)	1.70 (1.6, 1.8)	2,534 (20.43)	34,075 (20.63)	0.99 (0.9, 1.0)
NSAIDs	4,042 (50.90)	103,468 (60.98)	0.68 (0.6, 0.7)	8,179 (65.93)	99,331 (60.12)	1.26 (1.2, 1.3)
Antihypertensive drugs	4,670 (58.81)	91,832 (54.12)	1.20 (1.1, 1.3)	5,938 (47.86)	90,564 (54.82)	0.77 (0.7, 0.8)
COPD drugs	1,514 (19.07)	37,002 (21.81)	0.85 (0.8, 0.9)	2,373 (19.13)	36,143 (21.88)	0.85 (0.8, 0.9)
Opioids	3,284 (41.35)	73,501 (43.32)	0.93 (0.9, 1.0)	4,829 (38.92)	71,956 (43.55)	0.84 (0.8, 0.8)
Anti-thyroid drugs	60 (0.76)	1,548 (0.91)	0.83 (0.6, 1.1)	89 (0.72)	1,519 (0.92)	0.79 (0.6, 1.0)
**Healthcare utilization**,** 1 year prior**,** n (%)**						
No. of hospitalizations						
0	1,086 (13.68)	31,645 (18.65)	0.7 (0.6, 0.7)	3,091 (24.92)	29,640 (17.94)	1.47 (1.4, 1.5)
1	3,942 (49.64)	75,691 (44.61)	1.21 (1.1, 1.3)	5,293 (42.66)	74,340 (45.00)	0.92 (0.9, 1.0)
> 1	2,913 (36.68)	62,340 (36.74)	1.0 (0.9, 1.0)	4,022 (32.42)	61,231 (37.06)	0.83 (0.8, 0.9)
No. of outpatient visits						
0	447 (5.63)	11,503 (6.78)	0.83 (0.8, 0.9)	663 (5.34)	11,287 (6.83)	0.78 (0.7, 0.9)
1	458 (5.77)	22,329 (13.16)	0.42 (0.4, 0.5)	1,731 (13.95)	21,056 (12.74)	1.10 (1.1, 1.2)
> 1	7,036 (88.6)	135,844(80.06)	1.89 (1.8, 2.0)	10,012 (80.7)	132,868(80.42)	1.02 (1.0, 1.1)
No. of emergency visits						
0	6,858 (86.36)	142,861 (84.2)	1.18 (1.1, 1.3)	11,117 (89.61)	138,602(83.89)	1.61 (1.5, 1.7)
1	828 (10.43)	20,413 (12.03)	0.86 (0.8, 0.9)	1,049 (8.46)	20,192 (12.22)	0.68 (0.6, 0.7)
> 1	255 (3.21)	6,402 (3.77)	0.85 (0.8, 1.0)	240 (1.93)	6,417 (3.88)	0.51 (0.4, 0.6)

Values are n (%) unless otherwise specified

*As recorded in the Danish Hip Arthroplasty Registry

Abbreviation: n/a, not applicable. IQR, Interquartile range. COPD, Chronic obstructive pulmonary disease. NSAIDs, non-steroid anti-inflammatory drugs

The comorbidity burden, measured by the CCI, was similar between participants and non-participants before THA, whereas survey participants after THA were slightly healthier compared to non-participants (Fig. 2). The patterns of individual comorbidities, concomitant medication use, and healthcare utilization were reflective of the CCI pattern (Table 1).

For THA patients, prevalence ratios with corresponding 95% confidence intervals for participants and non-participants, both before and after surgery, aligned with the description provided above (Table 1).

Before THA, 34% had a low and 43% had a high level of education among survey participants, respectively, whereas 42% had a low and 15% had a high level of education among non-participants. The pattern of annual household income mirrored the distribution of educational levels. After THA, the distributions of educational levels and annual household income were more similar among survey participants and non-participants (Fig. 2).

### Description of KA surveys participants and non-participants

Among the 152,154 KA surgeries identified in the DKR, 10,575 (7.0%) patients participated in surveys before THA, and 9,400 (6.2%) participated in surveys after KA. The median time was 4.3 years from survey to KA among participants before KA and 4.2 years from surgery to survey among participants after KA.

**Fig. 3:**
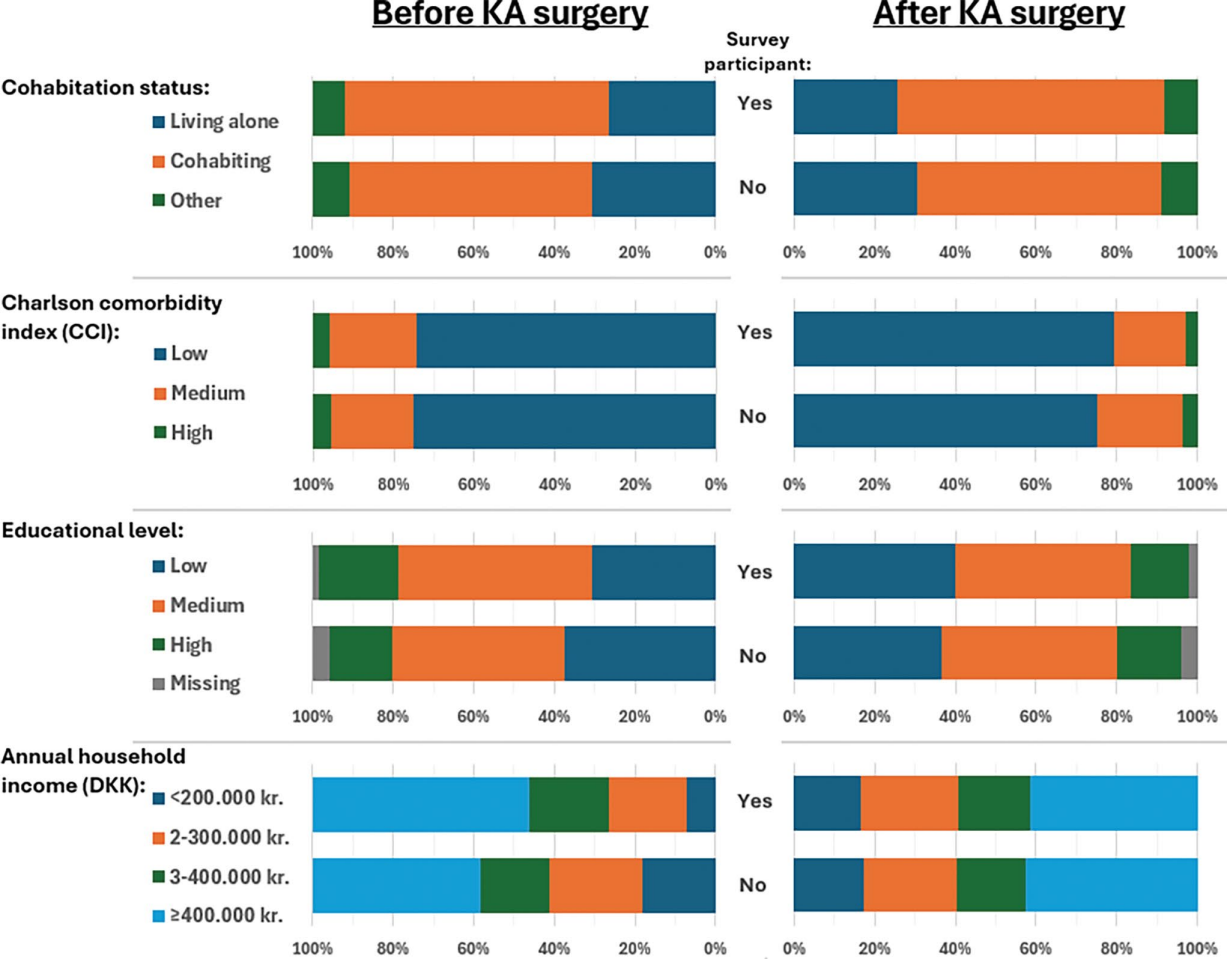
Prevalence and distribution of socioeconomic position markers and Charlson Comorbidity Index among knee arthroplasty patients (KA)* based on survey participation and the index surgery date. Exact numbers (n) and percentages (%) are provided in Supplementary Table 2. *As recorded in the Danish Knee Arthroplasty Registry. Abbreviation: DKK, The Danish krone

Both before and after KA, similar sex distribution and median age were observed among KA survey participants and non-participants (Table 2). Before KA, 27% of survey participants lived alone, compared to 30% for non-participants. After KA, 26% of survey participants lived alone, compared to 31% of non-participants (Fig. 3).

**Table 2:** Prevalence and prevalence ratios (PR) for various variables among knee arthroplasty patients (KA)* based on survey participation and the index surgery date

	Before KA surgery			After KA surgery		
	Survey participants (*n* = 10,575)	Non-survey participants (*n* = 141,579)	PR	Survey participants (*n* = 9,400)	Non-survey participants (*n* = 142,754)	PR
**Survey/surgery, years median**						
Survey to surgery	4.29	n/a	n/a	2.41	4.38	n/a
Surgery to survey	2.09	4.34	n/a	4.19	n/a	n/a
**Patient characteristics**						
Sex, n (%)						
Female	6,061 (57.31)	84,701 (59.83)	0.91 (0.9, 1.0)	5,509 (58.61)	85,253 (59.72)	0.96 (0.9, 1.0)
**Age**,** years median (IQR)**	70.19	68.65	n/a	67.11	68.9	n/a
Age group, n (%)						
35-<50	240 (2.27)	6,147 (4.34)	0.53 (0.5, 0.6)	407 (4.33)	5,980 (4.19)	1.03 (0.9, 1.2)
50-<55	527 (4.98)	8,971 (6.34)	0.79 (0.7, 0.9)	634 (6.74)	8,864 (6.21)	1.09 (1.0, 1.2)
55-<60	993 (9.39)	15,158 (10.71)	0.87 (0.8, 0.9)	1,118 (11.89)	15,033 (10.53)	1.14 (1.1, 1.2)
60-<65	1,417 (13.40)	21,732 (15.35)	0.86 (0.8, 0.9)	1,683 (17.90)	21,466 (15.04)	1.22 (1.2, 1.3)
65-<70	2,012 (19.03)	25,894 (18.29)	1.05 (1.0, 1.1)	2,051 (21.82)	25,855 (18.11)	1.24 (1.2, 1.3)
70-<75	2,360 (22.32)	27,161 (19.18)	1.19 (1.1, 1.3)	1,720 (18.30)	27,801 (19.47)	0.93 (0.9, 1.0)
75+	3,026 (28.61)	36,516 (25.79)	1.14 (1.1, 1.2)	1,787 (19.01)	37,755 (26.45)	0.67 (0.6, 0.7)
**Region of residence**, **n (%)**						
Capital	3,153 (29.82)	41,678 (29.44)	1.02 (1.0, 1.1)	2,867 (30.50)	41,964 (29.40)	1.06 (1.0, 1.1)
Zealand	1,646 (15.57)	25,850 (18.26)	0.84 (0.8, 0.9)	1,310 (13.94)	26,186 (18.34)	0.73 (0.7, 0.8)
Southern Denmark	2,436 (23.04)	31,376 (22.16)	1.05 (1.0, 1.1)	2,223 (23.65)	31,589 (22.13)	1.08 (1.0, 1.1)
Central Jutland	2,051 (19.39)	28,732 (20.29)	0.95 (0.9, 1.0)	1,773 (18.86)	29,010 (20.32)	0.92 (0.9, 1.0)
Northern Jutland	1,289 (12.19)	13,943 (9.85)	1.25 (1.2, 1.3)	1,227 (13.05)	14,005 (9.81)	1.35 (1.3, 1.4)
**Individual comorbidities**, **n (%)**						
COPD	402 (3.8)	5,431 (3.84)	0.99 (0.9, 1.1)	265 (2.82)	5,568 (3.9)	0.73 (0.6, 0.8)
Chronic renal impairment	107 (1.01)	1,290 (0.91)	1.1 (0.9, 1.3)	49 (0.52)	1,348 (0.94)	0.57 (0.4, 0.8)
Diabetes	1,402 (13.26)	17,332 (12.24)	1.09 (1.0, 1.2)	946 (10.06)	17,788 (12.46)	0.80 (0.7, 0.9)
Dementia	27 (0.26)	476 (0.34)	0.77 (0.5, 1.1)	28 (0.30)	475 (0.33)	0.90 (0.6, 1.4)
Any malignancy	1,541 (14.57)	15,137 (10.69)	1.39 (1.3, 1.5)	770 (8.19)	15,908 (11.14)	0.72 (0.7, 0.8)
Osteoporosis	538 (5.09)	5,433 (3.84)	1.31 (1.2, 1.4)	273 (2.90)	5,698 (3.99)	0.73 (0.6, 0.8)
**Concomitant medication**,** n (%)**						
Oral corticosteroids	1,112 (10.52)	17,770 (12.55)	1.23 (1.2, 1.3)	1,243 (13.22)	17,639 (12.36)	1.08 (1.0, 1.2)
Antiosteoporosis drugs	784 (7.41)	8,243 (5.82)	1.09 (1.0, 1.2)	412 (4.38)	8,615 (6.03)	0.73 (0.7, 0.8)
Anticoagulants	1,029 (9.73)	11,186 (7.90)	0.95 (0.9, 1.0)	583 (6.20)	11,632 (8.15)	0.76 (0.7, 0.8)
Antidiabetics	1,128 (10.67)	13,859 (9.79)	1.12 (1.1, 1.2)	758 (8.06)	14,229 (9.97)	0.8 (0.7, 0.9)
Antithrombotic	2,287 (21.63)	31,824 (22.48)	0.57 (0.2, 1.5)	2,144 (22.81)	31,967 (22.39)	1.02 (1.0, 1.1)
Hormone replacement therapy	1,559 (14.74)	18,723 (13.22)	0.63 (0.6, 0.7)	1,309 (13.93)	18,973 (13.29)	1.05 (1.0, 1.1)
Hormone deprivation therapy	< 5	100 (0.07)	0.65 (0.6, 0.8)	7 (0.07)	94 (0.07)	1.12 (0.5, 2.5)
Anxiolytics and sedatives	1,372 (12.97)	27,900 (19.71)	0.92 (0.9, 1.0)	1,857 (19.76)	27,415 (19.2)	1.03 (1.0, 1.1)
Antipsychotics	190 (1.80)	3,957 (2.79)	1.38 (1.3, 1.4)	197 (2.10)	3,950 (2.77)	0.76 (0.7, 0.9)
Antidepressants	1,308 (12.37)	18,862 (13.32)	0.72 (0.7, 0.8)	1,038 (11.04)	19,132 (13.4)	0.81 (0.8, 0.9)
Statins	3,684 (34.84)	38,903 (27.48)	1.05 (1.0, 1.1)	2,417 (25.71)	40,170 (28.14)	0.89 (0.8, 0.9)
Non-steroid anti-inflammatory drugs	5,714 (54.03)	88,554 (62.55)	1.12 (1.1, 1.2)	6,475 (68.88)	87,793 (61.50)	1.36 (1.3, 1.4)
Antihypertensive drugs	6,629 (62.69)	87,041 (61.48)	0.90 (0.9, 0.9)	5,623 (59.82)	88,047 (61.68)	0.93 (0.9, 1.0)
COPD drugs	1,380 (13.05)	16,649 (11.76)	0.73 (0.6, 0.9)	960 (10.21)	17,069 (11.96)	0.85 (0.8, 0.9)
Opioids	3,915 (37.02)	56,320 (39.78)	1.23 (1.2, 1.3)	3,568 (37.96)	56,667 (39.70)	0.93 (0.9, 1.0)
Anti-thyroid drugs	67 (0.63)	1,247 (0.88)	1.09 (1.0, 1.2)	74 (0.79)	1,240 (0.87)	0.91 (0.7, 1.2)
**Healthcare utilization**,** 1 year prior**,** n (%)**						
No. of hospitalizations						
0	4,495 (42.51)	46,031 (32.51)	1.49 (1.4, 1.5)	2,502 (26.62)	48,024 (33.64)	0.73 (0.7, 0.8)
1	3,372 (31.89)	52,067 (36.78)	0.82 (0.8, 0.9)	3,794 (40.36)	51,645 (36.18)	1.18 (1.1, 1.2)
>1	2,708 (25.61)	43,481 (30.71)	0.79 (0.7, 0.8)	3,104 (33.02)	43,085 (30.18)	1.13 (1.1, 1.2)
No. of outpatient visits						
0	2,732 (25.83)	18,902 (13.35)	2.1 (2.0, 2.2)	309 (3.29)	21,325 (14.94)	0.21 (0.2, 0.3)
1	441 (4.17)	8,422 (5.95)	0.70 (0.6, 0.8)	584 (6.21)	8,279 (5.80)	1.07 (1.0, 1.2)
>1	7,402 (70.00)	114,255 (80.70)	0.58 (0.5, 0.6)	8,507 (90.5)	113,150 (79.26)	2.39 (2.2, 2.6)
No. of emergency visits						
0	9,437 (89.24)	122,856 (86.78)	1.24 (1.2, 1.3)	8,147 (86.67)	124,146 (86.96)	0.98 (0.9, 1.0)
1	888 (8.40)	14,594 (10.31)	0.81 (0.8, 0.9)	1,001 (10.65)	14,481 (10.14)	1.05 (1.0, 1.1)
>1	250 (2.36)	4,129 (2.92)	0.82 (0.7, 0.9)	252 (2.68)	4,127 (2.89)	0.93 (0.8, 1.1)

Values are n (%) unless otherwise specified

*As recorded in the Danish Knee Arthroplasty Registry

Abbreviation: n/a, not applicable. IQR, Interquartile range. COPD, Chronic obstructive pulmonary disease. NSAIDs, non-steroid anti-inflammatory drugs

The comorbidity burden, measured by CCI, was nearly equally distributed between participants and non-participants before KA (Fig. 3). After KA, the survey participants seemed to have a slightly lower comorbidity burden than non-participants. The patterns of individual comorbidities, concomitant medication use, and healthcare utilization reflected the CCI (Table 2).

For KA patients, prevalence ratios with corresponding 95% confidence intervals for participants and non-participants, both before and after surgery, aligned with the above description (Table 2).

Before KA, 31% had a low and 20% had a high level of education among survey participants, respectively, whereas among non-participants, 37% had a low and 16% had a high level of education. The pattern of annual household income mirrored the distribution of educational levels. After KA, the distributions of academic levels and annual household income were similar among survey participants and non-participants (Fig. 3).

### Description of hip fracture survey participants and non-participants

Among the 92,600 hip fracture patients identified in the DMHFR, 3,557 (3.8%) patients participated in surveys before hip fracture surgery, and 2,056 (2.2%) participated in surveys after hip fracture surgery. The median time from survey to surgery among participants before hip fracture surgery was 3.8 years, and 2.6 years from surgery to survey among participants after hip fracture surgery.

**Fig. 4:**
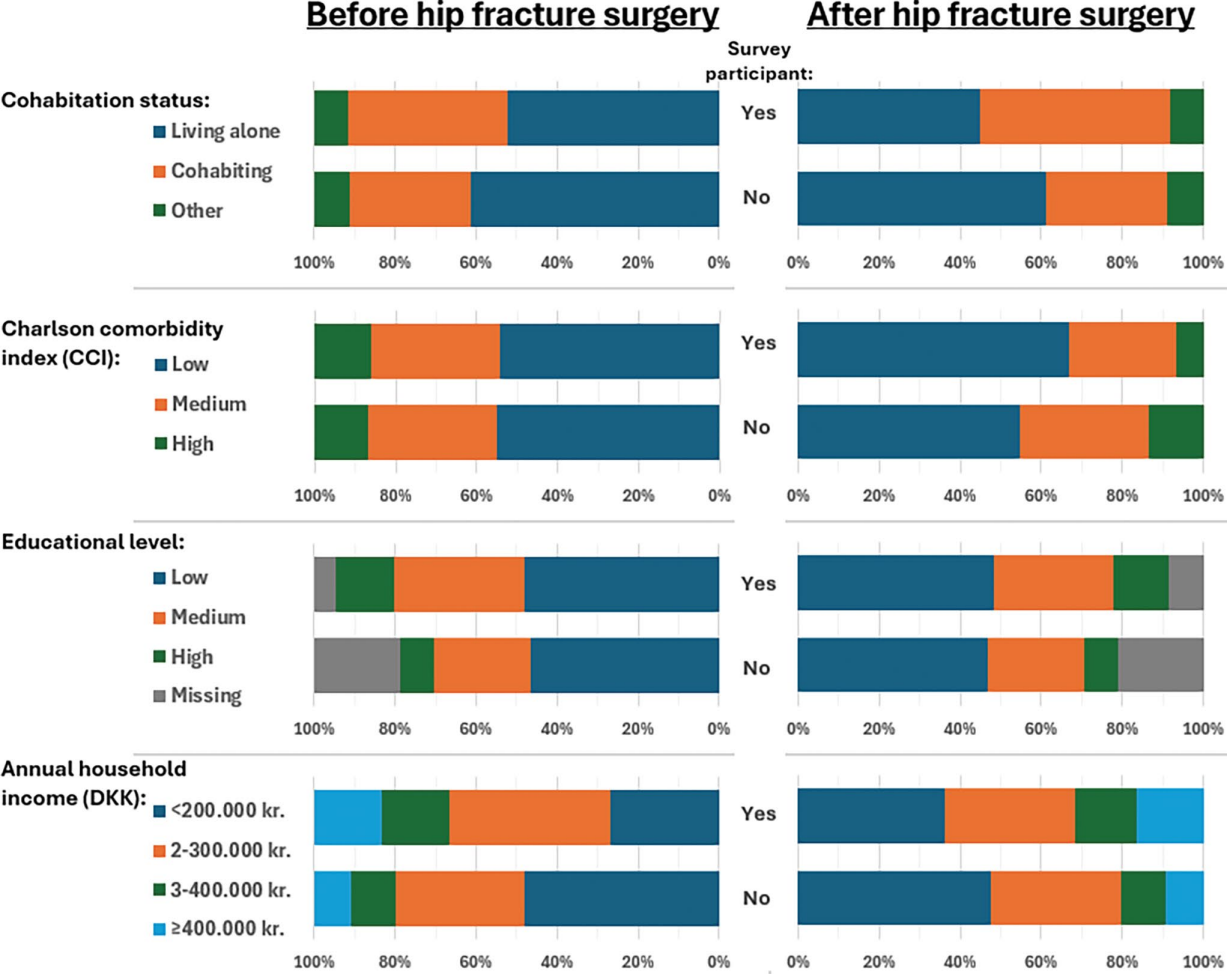
Prevalence and distribution of socioeconomic position markers and Charlson Comorbidity Index among hip fracture patients* based on survey participation and the index surgery date. Exact numbers (n) and percentages (%) are provided in Supplementary Table 3. *As recorded in the Danish Multidisciplinary Hip Fracture Registry (DMHFR). *DMFHR includes hip fracture patients at age 65 or older. Abbreviation: DKK, The Danish krone

Among survey participants before hip fracture, 66% were females, and the median age was 82 years, whereas among non-participants, 71% were females, and the median age was 83 years. After hip fracture, the prevalence of females among participants was 70% with a median age of 78. In contrast, the prevalence of females among non-participants was 71% with a median age of 83 years (Table 3). Before hip fracture, 52% of survey participants were living alone, compared to 61% of non-participants. After hip fracture, 45% of survey participants lived alone, whereas 61% of non-participants lived alone (Fig. 4).

**Table 3:** Prevalence and prevalence ratios (PR) for various variables among hip fracture patients* based on survey participation and the index surgery date

	Before hip fracture surgery			After hip fracture surgery		
	Survey participants (*n* = 3,557)	Non-survey participants (*n* = 89,043)	PR	Survey participants (*n* = 2,056)	Non-survey participants (*n* = 90,544)	PR
**Survey/surgery, ** **years median**						
Survey to surgery	3.77	n/a	n/a	2.77	3.81	n/a
Surgery to survey	1.62	2.74	n/a	2.64	n/a	n/a
**Patient characteristics**						
Sex, n (%)						
Female	2,350 (66.07)	63,463 (71.27)	0.79 (0.7, 0.9)	1,446 (70.33)	64,367 (71.09)	0.96 (0.9, 1.1)
Age, years median (IQR)	82.12	83.31	n/a	78.31	83.37	n/a
Age group, n* (%)						
35-<50	n/a	n/a	n/a	n/a	n/a	n/a
50-<55	n/a	n/a	n/a	n/a	n/a	n/a
55-<60	n/a	n/a	n/a	n/a	n/a	n/a
60-<65	n/a	n/a	n/a	n/a	n/a	n/a
65-<70	327 (9.19)	7211 (8.1)	1.14 (1.0, 1.3)	349 (16.97)	7,189 (7.94)	2.31 (2.1, 2.6)
70-<75	509 (14.31)	10,135 (11.38)	1.29 (1.2, 1.4)	379 (18.43)	10,265 (11.34)	1.74 (1.6, 1.9)
75+	2,721 (76.50)	71,697 (80.52)	0.80 (0.7, 0.9)	1,328 (64.59)	73,090 (80.72)	0.45 (0.4, 0.5)
**Region of residence**, **n (%)**						
Capital	886 (24.91)	22,589 (25.37)	0.98 (0.9, 1.1)	472 (22.96)	23,003 (25.41)	0.88 (0.8, 1.0)
Zealand	412 (11.58)	14,211 (15.96)	0.70 (0.6, 0.8)	236 (11.48)	14,387 (15.89)	0.69 (0.6, 0.8)
Southern Denmark	966 (27.16)	21,349 (23.98)	1.17 (1.1, 1.3)	630 (30.64)	21,685 (23.95)	1.39 (1.3, 1.5)
Central Jutland	733 (20.61)	20,116 (22.59)	0.89 (0.8, 1.0)	418 (20.33)	20,431 (22.56)	0.88 (0.8, 1.0)
Northern Jutland	560 (15.74)	10,778 (12.10)	1.34 (1.2, 1.5)	300 (14.59)	11,038 (12.19)	1.22 (1.1, 1.4)
**Individual comorbidities**, **n (%)**						
COPD	482 (13.55)	10,755 (12.08)	1.13 (1.0, 1.3)	185 (9.00)	11,052 (12.21)	0.72 (0.6, 0.8)
Chronic renal impairment	148 (4.16)	2,621 (2.94)	1.41 (1.2, 1.7)	30 (1.46)	2,739 (3.03)	0.48 (0.3, 0.7)
Diabetes	515 (14.48)	12,243 (13.75)	1.06 (1.0, 1.2)	267 (12.99)	12,491 (13.80)	0.93 (0.8, 1.1)
Dementia	321 (9.02)	11,736 (13.18)	0.66 (0.6, 0.7)	50 (2.43)	12,007 (13.26)	0.17 (0.1, 0.2)
Any malignancy	858 (24.12)	16,447 (18.47)	1.38 (1.3, 1.5)	277 (13.47)	17,028 (18.81)	0.68 (0.6, 0.8)
Osteoporosis	731 (20.55)	15,084 (16.94)	1.26 (1.2, 1.4)	281 (13.67)	15,534 (17.16)	0.77 (0.7, 0.9)
**Concomitant medication**,** n (%)**						
Oral corticosteroids	393 (11.05)	9,298 (10.44)	1.06 (1.0, 1.2)	187 (9.10)	9,504 (10.5)	0.86 (0.7, 1.0)
Antiosteoporosis drugs	712 (20.02)	14,933 (16.77)	1.23 (1.1, 1.3)	326 (15.86)	15,319 (16.92)	0.93 (0.8, 1.0)
Anticoagulants	597 (16.78)	8,926 (10.02)	1.76 (1.6, 1.9)	157 (7.64)	9,366 (10.34)	0.72 (0.6, 0.9)
Antidiabetics	362 (10.18)	9,159 (10.29)	0.99 (0.9, 1.1)	214 (10.41)	9,307 (10.28)	1.01 (0.9, 1.2)
Antithrombotic	1,365 (38.38)	37,583 (42.21)	0.86 (0.8, 0.9)	772 (37.55)	38,176 (42.16)	0.83 (0.8, 0.9)
Hormone replacement therapy	387 (10.88)	7478 (8.4)	1.32 (1.2, 1.5)	247 (12.01)	7618 (8.41)	1.47 (1.3, 1.7)
Hormone deprivation therapy	12 (0.34)	327 (0.37)	0.92 (0.5, 1.6)	< 5	339 (0.37)	0 (.,.)
Anxiolytics and sedatives	823 (23.14)	27,205 (30.55)	0.69 (0.6, 0.8)	543 (26.41)	27,485 (30.36)	0.83 (0.8, 0.9)
Antipsychotics	216 (6.07)	8,547 (9.60)	0.62 (0.5, 0.7)	84 (4.09)	8,679 (9.59)	0.41 (0.3, 0.5)
Antidepressants	924 (25.98)	28,553 (32.07)	0.75 (0.7, 0.8)	427 (20.77)	29,050 (32.08)	0.56 (0.5, 0.6)
Statins	1,161 (32.64)	21,473 (24.12)	1.50 (1.4, 1.6)	609 (29.62)	22,025 (24.33)	1.30 (1.2, 1.4)
Non-steroid anti-inflammatory drugs	683 (19.2)	20,009 (22.47)	0.83 (0.8, 0.9)	549 (26.70)	20,143 (22.25)	1.27 (1.2, 1.4)
Antihypertensive drugs	2,529 (71.1)	61,665 (69.25)	1.09 (1.0, 1.2)	1,315 (63.96)	62,879 (69.45)	0.79 (0.7, 0.9)
COPD drugs	896 (25.19)	20,634 (23.17)	1.11 (1.0, 1.2)	454 (22.08)	21,076 (23.28)	0.94 (0.8, 1.0)
Opioids	1,109 (31.18)	28,332 (31.82)	0.97 (0.9, 1.0)	496 (24.12)	28,945 (31.97)	0.68 (0.6, 0.8)
Anti-thyroid drugs	77 (2.16)	2,204 (2.48)	0.88 (0.7, 1.1)	46 (2.24)	2,235 (2.47)	0.91 (0.7, 1.2)
**Healthcare utilization**,** 1 year prior**,** n (%)**						
No. of hospitalizations						
0	< 5	60 (0.07)	0 (.,.)	< 5	60 (0.07)	0.75 (0.1, 5.2)
1	2,343 (65.87)	58,180 (65.34)	1.02 (1.0, 1.1)	1,547 (75.28)	58,976 (65.13)	1.61 (1.5, 1.8)
>1	1,214 (34.13)	30,803 (34.59)	0.98 (0.9, 1.1)	508 (24.72)	31,509 (34.80)	0.62 (0.6, 0.7)
No. of outpatient visits						
0	1,657 (46.58)	47,904 (53.80)	0.76 (0.7, 0.8)	1,101 (53.55)	48,460 (53.52)	1.00 (0.9, 1.1)
1	456 (12.82)	10,905 (12.25)	1.05 (1.0, 1.2)	230 (11.19)	11,131 (12.29)	0.90 (0.8, 1.0)
>1	1,444 (40.6)	30,234 (33.95)	1.31 (1.2, 1.4)	725 (35.26)	30,953 (34.19)	1.05 (1.0, 1.2)
No. of emergency visits						
0	998 (28.06)	22,007 (24.72)	1.18 (1.1, 1.3)	546 (26.56)	22,459 (24.80)	1.09 (1.0, 1.2)
1	1,912 (53.75)	51,377 (57.70)	0.86 (0.8, 0.9)	1,289 (62.69)	52,000 (57.43)	1.24 (1.1, 1.4)
>1	647 (18.19)	15,659 (17.59)	1.04 (1.0, 1.1)	221 (10.75)	16,085 (17.76)	0.56 (0.5, 0.7)

Values are n (%) unless otherwise specified

*As recorded in the Danish Multidisciplinary Hip Fracture Registry (DMHFR)

*DMFHR includes hip fracture patients at age 65 or older

Abbreviation: n/a, not applicable. IQR, Interquartile range. COPD, Chronic obstructive pulmonary disease. NSAIDs, non-steroid anti-inflammatory drugs

The comorbidity burden before hip fracture was nearly equally distributed between participants and non-participants (Fig. 4). After hip fracture, a lower CCI score was observed among the survey participants compared to both the non-participants and the pre-surgery data. Upon examining individual comorbidities and concomitant medication use, differences were observed between participants and non-participants in the prevalence of dementia, any malignancy, osteoporosis, anticoagulants, antithrombotic, anxiolytics, sedatives, antidepressants, and statins (Table 3).

For hip fracture patients, prevalence ratios with corresponding 95% confidence intervals for participants and non-participants, both before and after surgery, aligned with the description above (Table 3).

Among survey participants before hip fracture, 48% had low education and 14% had high education, whereas among non-participants, 47% had low education and 8% had high education. The income was higher among participants than non-participants before the hip fracture surgery. Similar distributions were observed after hip fracture surgery (Fig. 4).

## Discussion

This study aimed to examine whether participants in the Danish National Health Survey in 2010, 2013, 2017, and 2021 were representative of all patients undergoing THA, KA, and hip fracture surgery as registered in the DHR, DKR, and DMHFR. We found that the survey participants provided a sample largely representative of all THA, KA, and hip fracture patients. However, survey participants had slightly better health and social status than non-participants. Survey data could play a valuable role in clinical research when used as a supplement to orthopedic registries. Employing robust epidemiological designs is essential for minimizing observed discrepancies and overcoming methodological challenges in cohort studies.

### Implication and perspectives

The utilization of PROM data has been in increasing demand within orthopedic research. This trend is attributed to the significant impact that PROM data exerts on current and prospective health policy decisions [13, 25, 26]. Orthopedic registries have provided important knowledge on risk factors associated with THA, KA, and hip fracture incidence and prognosis [27–29]. However, data on relevant exposures, confounders, and outcomes such as self-rated health risk behaviors, quality of life, morbidity, and social relations are rarely captured in orthopedic registries. Including this data and information could serve as a valuable tool for, i.e., improving surgeons’ ability to identify patients with poor prognosis and general risk assessment [30]. Instead of expanding the orthopedic registries to include the collection of patient-reported data and thereby increasing registration burden and costs in the healthcare system, available comprehensive survey data could be a valuable alternative. While it is crucial to validate surveys before use to uncover any selection and information bias, validation studies are rarely performed [31, 32]. This study is the first to provide a comprehensive comparison of survey participants and non-participants in the Danish Nationale Health Survey, both before and after surgery, on multiple patient characteristics to validate the potential use of data from this survey for orthopedic research among THA, KA and hip fracture patients. The findings could aid researchers in planning and designing future studies based on a combination of orthopedic and survey data [33].

The survey data could be used to identify new risk factors for sustaining THA, KA, or hip fractures. A previous study has reported that self-reported perceived stress as registered in the Danish National Health Survey is associated with an increased risk of any osteoporotic fracture, in particular risk of hip fracture [34]. In continuation, social isolation and social networks have a large impact on the risk of developing numerous diseases and conditions [35–37]. Evidence is sparse on the impact of these factors on sustaining THA, KA, or hip fracture.

The survey data on health risk behaviors can be used as important confounders in future studies on the associations between exposures and outcomes in THA, KA, and hip fracture patients. The survey data provide a unique source of data on often unmeasured confounders [38]. However, using survey data to assess confounding variables can present methodological challenges, as the survey participants appear slightly healthier than non-participants. In this case, a variable such as smoking may introduce residual confounding, potentially leading to an underestimation of the true effect in a study [39]. Conversely, applying calibrated non-response weighting to health survey data can mitigate non-response bias to some extent [40].

Finally, the survey data could be a valuable tool for studying the prognosis of THA, KA, or hip fracture patients, as demonstrated in other areas of research [41]. Preliminary data show that self-reported health and stress, as registered in the Danish National Health Survey, are independently associated with increased use of opioids after THA [42, 43].

### Strengths and limitations

A general strength of the Danish National Health Surveys is that they are based on a large national random sample of individuals in the general population setting. Furthermore, a strength is the use of a broad range of questions included in the questionnaire [14]. However, the surveys rely solely on questionnaires without incorporating any objective measures [44, 45].

The response rate was 59.5%, 54.0%, and 58.7% in the 2010, 2013, and 2017 survey waves respectively [14]. Overall, the response rates were lower among young men and old women and among individuals who were unmarried and had low sociodemographic status [14], which is also reflected in our results. Furthermore, as mentioned in the method section, we lack information regarding whether THA, KA, and hip fracture non-participants in the surveys were not invited or did not respond [46, 47]. Individuals who do not respond cannot be expected to be distributed at random [15]. However, using weights to minimize the impact of non-response can be applied to mitigate potential issues in generalizing the results of future studies [40], thereby reducing the degree of non-response bias and enhancing the study validity [46, 47]. It is important to emphasize the need for appropriate study designs and careful consideration of statistical methods when analyzing health survey data. While random sampling and using weights can somewhat reduce bias, previous research indicates that certain patient groups may remain slightly underrepresented [48].

Furthermore, the number of participants may pose challenges depending on the study and research setting. For instance, survey participants after hip fracture surgery constitute a small sample size, which can reduce a study’s power, thus increasing the risk of random error [49].

The Danish National Health Surveys are not designed for a specific study population. Using this general health questionnaire among THA, KA, or hip fracture patients can reduce information bias including social desirability bias [50]. Cross-linking the questionnaire to specific orthopedic cohorts ensures that the research setting does not significantly influence patients’ responses [47, 50, 51].

In conducting research with elderly orthopedic surgery patients, it is necessary to consider death as a competing risk [52]. Since we could only calculate prevalences, the survey participants could exemplify the ‘healthy survivor bias’ [53, 54]. This bias could account for some of the observed differences in our study, particularly in patients with hip fractures.

## Conclusion

Data from the Danish National Health Surveys provided a sample that appeared to be largely representative of the entire THA, KA, and hip fracture patients in Denmark aged 35 years or older based on several patient and healthcare utilization characteristics. However, survey participants had slightly better health and social status than non-participants. Consequently, the survey data could be a valuable source for further studies of the risks and outcomes associated with patients undergoing THA and KA and those suffering from hip fractures. It is, however, important to consider the outlined similarities and differences between participants and non-participants when analyzing and interpreting survey data.

## Electronic supplementary material

Below is the link to the electronic supplementary material.

Supplementary Material 1

## Data Availability

The data analysed in this study were made available to us in an anonymized form on the servers of Statistics Denmark. Thus, the data are not free to be shared, and access may be granted by relevant authorities in Denmark.
