# Supplementary material for: Representativeness of participants in the Danish National Health Survey across 422,371 orthopedic surgeries: a study of hip and knee arthroplasty and hip fracture patients
Source: Arch Orthop Trauma Surg. 2025 May 27;145(1):319. doi: 10.1007/s00402-025-05924-7 (PMC12116827; doi:10.1007/s00402-025-05924-7)
Supplement: Supplementary file 1 — Supplementary Material 1 [file 402_2025_5924_MOESM1_ESM.docx]

**Online Supplementary Material**

**Journal**

Archives of Orthopaedic and Trauma Surgery

**Article title**

Representativeness of Participants in the Danish National Health Survey Across 422,371 Orthopedic Surgeries: A Study of Hip and Knee Arthroplasty and Hip Fracture Patients

**Authors and affiliations**

Blinded ref. (<https://link.springer.com/journal/402/submission-guidelines>)

**Corresponding Author**

Blinded ref. (<https://link.springer.com/journal/402/submission-guidelines>)

| **Supplementary Table 1: Prevalence and prevalence ratios (PR) for socioeconomic position markers and Charlson Comorbidity Index among hip arthroplasty patients (THA)* based on survey participation and the index surgery date.** | | | | | | |
| --- | --- | --- | --- | --- | --- | --- |
|  | **Before THA surgery** | | | **After THA surgery** | | |
|  | **Survey participants (n=7,941)** | **Non-survey participants (n=169,676)** | **PR**  **(95% CI)** | **Survey participants (n=12,406)** | **Non-survey participants (n=165,211)** | **PR**  **(95% CI)** |
| **Cohabitation status,**  **n (%)** |  |  |  |  |  |  |
| Living alone | 2,402 (30.25) | 59,101 (34.83) | 0.82 (0.8, 0.9) | 3,283 (26.46) | 58,220 (35.24) | 0.68 (0.6, 0.7) |
| Cohabiting | 4,939 (62.20) | 95,314 (56.17) | 1.27 (1.2, 1.3) | 8,114 (65.4) | 92,139 (55.77) | 1.46 (1.4, 1.5) |
| Other | 600 (7.56) | 15,261 (8.99) | 0.83 (0.8, 0.9) | 1,009 (8.13) | 14,852 (8.99) | 0.90 (0.8, 1.0) |
| **Charlson Comorbidity**  **index (CCI),**  **n (%)** |  |  |  |  |  |  |
| Low | 5,891 (74.18) | 129,446 (76.29) | 0.9 (0.85, 0.95) | 10,273 (82.81) | 125,064 (75.7) | 1.50 (1.4, 1.6) |
| Medium | 1,671 (21.04) | 32,856 (19.36) | 1.1 (1.04, 1.17) | 1,849 (14.90) | 32,678 (19.78) | 0.73 (0.7, 0.8) |
| High | 379 (4.77) | 7,374 (4.35) | 1.1 (0.98, 1.22) | 284 (2.29) | 7,469 (4.52) | 0.51 (0.5, 0.6) |
| **Educational level,**  **n (%)** |  |  |  |  |  |  |
| Low | 2,735 (34.44) | 70,441 (41.52) | 0.75 (0.7, 0.8) | 5,121 (41.28) | 68,055 (41.19) | 1,00 (0.9, 1.0) |
| Medium | 3,377 (42.53) | 60,256 (35.51) | 1.33 (1.3, 1.4) | 4,790 (38.61) | 58,843 (35.62) | 1.13 (1.1, 1.2) |
| High | 1,714 (21.58) | 25,618 (15.1) | 1.51 (1.4, 1.6) | 2,171 (17.50) | 25,161 (15.23) | 1.17 (1.1, 1.2) |
| Missing | 115 (1.45) | 13,361 (7.87) | 0.18 (0.1, 0.2) | 324 (2.61) | 13,152 (7.96) | 0.33 (0.3, 0.4) |
| **Annual household**  **Income (DKK),**  **n (%)** |  |  |  |  |  |  |
| <200.000 kr. | 798 (10.05) | 46,710 (27.53) | 0.31 (0.3, 0.4) | 2,402 (19.36) | 45,106 (27.3) | 0.66 (0.6, 0.7) |
| 200.000-300.000 kr. | 1,869 (23.54) | 41,367 (24.38) | 0.96 (0.9, 1.0) | 2,811 (22.66) | 40,425 (24.47) | 0.91 (0.9, 1.0) |
| 300.000-400.000 kr. | 1,510 (19.02) | 25,927 (15.28) | 1.29 (1.2, 1.4) | 2,145 (17.29) | 25,292 (15.31) | 1.14 (1.1, 1.2) |
| ≥400.000 kr. | 3,764 (47.40) | 55,672 (32.81) | 1.79 (1.7, 1.9) | 5,048 (40.69) | 54,388 (32.92) | 1.36 (1.3, 1.4) |
| Values are n (%) unless otherwise specified. *As recorded in the Danish Hip Arthroplasty Registry  Abbreviation: DKK, The Danish krone. | | | | | | |

| **Supplementary Table 2: Prevalence and prevalence ratios (PR) for socioeconomic position markers and Charlson Comorbidity Index among knee arthroplasty patients (KA)* based on survey participation and the index surgery date.** | | | | | | |
| --- | --- | --- | --- | --- | --- | --- |
|  | **Before KA surgery** | | | **After KA surgery** | | |
|  | **Survey participants (n=10,575)** | **Non-survey participants (n=141,579)** | **PR** | **Survey participants (n=9,400)** | **Non-survey participants (n=142,754)** | **PR** |
| **Cohabitation status,**  **n (%)** |  |  |  |  |  |  |
| Living alone | 2,802 (26.50) | 43,145 (30.47) | 0.83 (0.8, 0.9) | 2,407 (25.61) | 43,540 (30.50) | 0.80 (0.8, 0.8) |
| Cohabiting | 6,921 (65.45) | 85,527 (60.41) | 1.22 (1.2, 1.3) | 6,212 (66.09) | 86,236 (60.41) | 1.26 (1.2, 1.3) |
| Other | 852 (8.06) | 12,907 (9.12) | 0.88 (0.8, 1.0) | 781 (8.31) | 12,978 (9.09) | 0.91 (0.8, 1.0) |
| **Charlson Comorbidity**  **index (CCI),**  **n (%)** |  |  |  |  |  |  |
| Low | 7,879 (74.51) | 107,055(75.62) | 0.95 (0.9, 1.0) | 7,471 (79.48) | 107,463(75.28) | 1.25 (1.2, 1.3) |
| Medium | 2,287 (21.63) | 29,300 (20.7) | 1.05 (1.0, 1.1) | 1,672 (17.79) | 29,915 (20.96) | 0.83 (0.8, 0.9) |
| High | 409 (3.87) | 5,224 (3.69) | 1.05 (0.9, 1.2) | 257 (2.73) | 5,376 (3.77) | 0.73 (0.6, 0.8) |
| **Educational level,**  **n (%)** |  |  |  |  |  |  |
| Low | 3,238 (30.62) | 52,928 (37.38) | 0.75 (0.7, 0.8) | 3,742 (39.81) | 52,424 (36.72) | 1.13 (1.1, 1.2) |
| Medium | 5,090 (48.13) | 60,725 (42.89) | 1.22 (1.2, 1.3) | 4,093 (43.54) | 61,722 (43.24) | 1.01 (1.0, 1.1) |
| High | 2,093 (19.79) | 21,961 (15.51) | 1.31 (1.3, 1.4) | 1,365 (14.52) | 22,689 (15.89) | 0.90 (0.9, 1.0) |
| Missing | 154 (1.46) | 5,965 (4.21) | 0.35 (0.3, 0.4) | 200 (2.13) | 5,919 (4.15) | 0.52 (0.4, 0.6) |
| **Annual household**  **Income (DKK), n (%)** |  |  |  |  |  |  |
| <200.000 kr. | 737 (6.97) | 25,641 (18.11) | 0.36 (0.3, 0.4) | 1,566 (16.66) | 24,812 (17.38) | 0.95 (0.9, 1.0) |
| 200.000-300.000 kr. | 2,080 (19.67) | 32,702 (23.10) | 0.83 (0.8, 0.9) | 2,252 (23.96) | 32,530 (22.79) | 1.06 (1.0, 1.1) |
| 300.000-400.000 kr. | 2,066 (19.54) | 24,169 (17.07) | 1.17 (1.1, 1.2) | 1,674 (17.81) | 24,561 (17.21) | 1.04 (1.0, 1.1) |
| ≥400.000 kr. | 5,692 (53.83) | 59,067 (41.72) | 1.57 (1.5, 1.6) | 3,908 (41.57) | 60,851 (42.63) | 0.96 (0.9, 1.0) |
| Values are n (%) unless otherwise specified.  *As recorded in the Danish Knee Arthroplasty Registry  Abbreviation: DKK, The Danish krone. | | | | | | |

| **Supplementary Table 3: Prevalence and prevalence ratios (PR) for socioeconomic position markers and Charlson Comorbidity Index among hip fracture patients* based on survey participation and the index surgery date.** | | | | | | |
| --- | --- | --- | --- | --- | --- | --- |
|  | **Before hip fracture surgery** | | | **After hip fracture surgery** | | |
|  | **Survey participants (n=3,557)** | **Non-survey participants (n=89,043)** | **PR** | **Survey participants (n=2,056)** | **Non-survey participants (n=90,544)** | **PR** |
| **Cohabitation status,**  **n (%)** |  |  |  |  |  |  |
| Living alone | 1,851 (52.04) | 54,418 (61.11) | 0.70 (0.7, 0.8) | 926 (45.04) | 55,343 (61.12) | 0.53 (0.5, 0.6) |
| Cohabiting | 1,406 (39.53) | 26,760 (30.05) | 1.50 (1.4, 1.6) | 962 (46.79) | 27,204 (30.05) | 2.01 (1.9, 2.2) |
| Other | 300 (8.43) | 7,865 (8.83) | 0.95 (0.9, 1.1) | 168 (8.17) | 7,997 (8.83) | 0.92 (0.8, 1.1) |
| **Charlson Comorbidity**  **index (CCI),**  **n (%)** |  |  |  |  |  |  |
| Low | 1,929 (54.23) | 49,072 (55.11) | 0.97 (0.9, 1.0) | 1,376 (66.93) | 49,625 (54.81) | 1.65 (1.5, 1.8) |
| Medium | 1,128 (31.71) | 28,208 (31.68) | 1.00 (0.9, 1.1) | 543 (26.41) | 28,793 (31.80) | 0.77 (0.7, 0.9) |
| High | 500 (14.06) | 11,763 (13.21) | 1.07 (1.0, 1.2) | 137 (6.66) | 12,126 (13.39) | 0.47 (0.4, 0.6) |
| **Educational level,**  **n (%)** |  |  |  |  |  |  |
| Low | 1,708 (48.02) | 41,528 (46.64) | 1.05 (1.0, 1.1) | 990 (48.15) | 42,246 (46.66) | 1.06 (1.0, 1.2) |
| Medium | 1,151 (32.36) | 20,999 (23.58) | 1.52 (1.4, 1.6) | 612 (29.77) | 21,538 (23.79) | 1.35 (1.2, 1.5) |
| High | 501 (14.08) | 7,515 (8.44) | 1.73 (1.6, 1.9) | 281 (13.67) | 7,735 (8.54) | 1.67 (1.5, 1.9) |
| Missing | 197 (5.54) | 19,001 (21.34) | 0.22 (0.2, 0.3) | 173 (8.41) | 19,025 (21.01) | 0.35 (0.3, 0.4) |
| **Annual household**  **Income (DKK),**  **n (%)** |  |  |  |  |  |  |
| <200.000 kr. | 959 (26.96) | 42,766 (48.03) | 0.41 (0.4, 0.5) | 744 (36.19) | 42,981 (47.47) | 0.63 (0.6, 0.7) |
| 200.000-300.000 kr. | 1,407 (39.56) | 28,493 (32.00) | 1.37 (1.3, 1.5) | 665 (32.34) | 29,235 (32.29) | 1.00 (0.9, 1.1) |
| 300.000-400.000 kr. | 595 (16.73) | 9,585 (10.76) | 1.63 (1.5, 1.8) | 310 (15.08) | 9,870 (10.90) | 1.44 (1.3, 1.6) |
| ≥400.000 kr. | 596 (16.76) | 8,199 (9.21) | 1.92 (1.8, 2.1) | 337 (16.39) | 8,458 (9.34) | 1.87 (1.7, 2.1) |
| Values are n (%) unless otherwise specified.  *As recorded in the Danish Multidisciplinary Hip Fracture Registry (DMHFR)  *DMFHR includes hip fracture patients at age 65 or older. Abbreviation: DKK, The Danish krone. | | | | | | |

**Appendix Table A** – Individual comorbidities, Charlson Comorbidity Index (CCI), and Concomitant medication, by International Classification of Disease 10th Edition (ICD‐10), Anatomical Therapeutic Chemical Classification System (ATC), and Procedure codes

| Category | ICD-10 Codes | ATC Codes | Procedure Codes |
| --- | --- | --- | --- |
| Individual comorbidities | | | |
| Chronic obstructive pulmonary disease (COPD) | J44 |  |  |
| Chronic renal impairment | N18 |  | BJFD2 |
| Diabetes | E10-E14, O24 (except O244), H360, G632, H360, N083 | A10B, A10A |  |
| Dementia | F00-F03, F051, G30, G231, G311, G318B, G318E |  |  |
| Any malignancy | C00-C96 |  |  |
| Osteoporosis | M80, M81, M82 |  |  |
| Charlson Comorbidity Index (CCI) | | | |
| Myocardial infarction | I21; I22; I23 |  |  |
| Chronic pulmonary disease | J70.1; J70.3; J84.1;  J92.0; J96.1; J98.2;  J98.3 |  |  |
| Connective tissue disease | M05; M06; M08; M09;  M30; M31; M32; M33;  M34; M35; M36; D86 |  |  |
| Congestive heart failure | I50; I11.0; I13.0; I13.2 |  |  |
| Peripheral vascular disease | I70; I71; I72; I73; I74;  I77 |  |  |
| Cerebrovascular disease | I60-I69; G45; G46 |  |  |
| Dementia | F00-F03; F05.1; G30 |  |  |
| Ulcer disease | K22.1; K25-K28 |  |  |
| Mild liver disease | B18; K70.0-K70.3;  K70.9; K71; K73; K74;  K76.0 |  |  |
| Diabetes, uncomplicated | E10.0, E10.1; E10.9,  E11.0; E11.1; E11.9 |  |  |
| Diabetes, complicated | E10.2-E10.8 E11.2-  E11.8 |  |  |
| Hemiplegia | G81; G82 |  |  |
| Moderate to severe renal disease | I12, I13, N00-N05, N07,  N11; N14; N17-N19;  Q61 |  |  |
| Any solid tumor | C00-C75 |  |  |
| Leukemia | C91-C95 |  |  |
| Lymphoma | C81-C85; C88; C90; C96 |  |  |
| Moderate or severe liver disease | B15.0, B16.0, B16.2,  B19.0; K70.4; K72;  K76.6; I85 |  |  |
| Metastatic solid tumor | C76-C80 |  |  |
| HIV/AIDS | B21-B24 |  |  |
| Concomitant medication | | | |
| Oral corticosteroids |  | H02 |  |
| Antiosteoporosis drugs |  | M05BA04, M05BB03, M05BX04, M05BA01,  M05BA06, H05AA03, G03XC01, M05BA07, M05BX03, H05AA02, M05BA08 |  |
| Anticoagulants |  | B01AA, B01AE, B01AF, B01AB, B01AX |  |
| Antidiabetics |  | A10 |  |
| Antithrombotic |  | B01AC, N02BA01, N02BA51 |  |
| Hormone replacement therapy |  | G03C, G03D, G03F, L02AA |  |
| Hormone deprivation therapy |  | G03H, L02BA, L02BG |  |
| Anxiolytics and sedatives |  | N05B, N05C |  |
| Antipsychotics |  | N05A |  |
| Antidepressants |  | N06A |  |
| Statins |  | C10AA |  |
| Non-steroid anti-inflammatory drugs |  | M01A |  |
| Antihypertensive drugs |  | C02, C03, C04, C07, C08, C09 |  |
| COPD drugs |  | R03, R05, R07A |  |
| Opioids |  | N02A |  |
| Anti-thyroid drugs |  | H03B |  |
